# Three-Dimensional Assessment of Temporomandibular Joint Morphology and Facial Asymmetry in Individuals with Different Vertical Skeletal Growth Patterns

**DOI:** 10.3390/ijerph20021437

**Published:** 2023-01-12

**Authors:** Rohan Diwakar, Rosaria Bucci, Ankur Kaushik, Anubhav Bansal, Paolo Bucci, Anuraj Singh Kochhar, Gianrico Spagnuolo

**Affiliations:** 1 Department of Orthodontics, PDM Dental College, Bahadurgarh 124507, India; 2 Department of Neurosciences, Reproductive Sciences and Oral Sciences, University of Naples Federico II, 80131 Naples, Italy; 3 Department of Public Health, University of Naples Federico II, 80131 Naples, Italy; 4 Faculty of Dentistry, University of Toronto, Toronto, ON M5G 0C1, Canada; 5 Therapeutic Dentistry Department, Institute for Dentistry, Sechenov University, 119991 Moscow, Russia

**Keywords:** temporomandibular joint, mandibular condyle, cone-beam computed tomography, facial asymmetry, imaging, three-dimensional

## Abstract

The aim of the current study was to investigate, by means of Cone-Beam Computed Tomography (CBCT), condyle–fossa relationship, temporomandibular joint (TMJ) morphology and facial asymmetry in subjects with different vertical skeletal growth patterns. CBCT of 56 patients (112 TMJs) were categorized into three groups according to the mandibular plane angle (MP): Hypodivergent (MP ≤ 23°), Normodivergent (23° < MP < 30°), and Hyperdivergent (MP ≥ 30°). TMJ spaces, width and depth of the condyle and thickness of the fossa were measured. Horizontal and vertical measurements were used to assess facial asymmetry. One-way Analysis of Variance (ANOVA) and post-hoc Turkey tests were computed for the between-groups comparison. Statistical significance was set at *p* < 0.05. Larger anterior joint space and smaller condylar dimensions (medio-lateral diameter and medio-lateral thickness) were observed in the hyperdivergent group compared to the normodivergent and hypodivergent groups. Right condylar distances to midsagittal plane were significantly larger than left distances in all the three groups. A vertical pattern of growth in healthy individuals seems to be associated with condylar position and dimension, while facial asymmetry values do not differ among different vertical groups.

## 1. Introduction

The appearance of the face is an important criterion for positive interpersonal communication. Side-to-side symmetry of the face plays a crucial role in the overall facial attractiveness. Although minor facial asymmetries are recognized as normal, severe asymmetry of the craniofacial structures is not well tolerated by patients. In fact, there is an increasing number of individuals seeking orthodontic evaluation and orthognathic surgical approaches, in order to correct facial discrepancies [1].

Facial asymmetry can be defined as any discrepancy in shape and size of one side of the face compared to the opposite side. This discrepancy may be due to genetic unbalanced growth of maxillofacial structures, but also to environmental factors such as fractures, tumors, and dimensional alteration of soft tissues [2]. Studies have supported that facial asymmetry not only affects dental occlusion and maxillo-mandibular dimensions, but it can also affect the morphology of the Temporomandibular Joint (TMJ) and the condylar position in the glenoid fossa [3,4]. Furthermore, recent studies supported that condylar shape and dimension, and condyle–fossa relationship are also influenced by diverse sagittal and vertical facial features [5,6,7]. In particular, hyperdivergent subjects have smaller condyles with higher antero-posterior inclination angles as compared to hypodivergent individuals. In addition, posterior condylar position is more frequently observed in low-angle individuals, whereas anterior condylar position is more prevalent in high-angle subjects [6,7].

A comprehensive radiographic analysis is required to assess condyle–fossa relationship and TMJ morphology. For many years, conventional bi-dimensional imaging techniques, such as posteroanterior cephalograms, submentovertex views, and panoramic views, have been used for diagnostic purposes [4]. However, 2D films suffer from several limitations caused by head posture, overlap of anatomical structures, magnification and distortion. Hence, bi-dimensional images are not able to provide adequate information regarding TMJ features. The introduction of 3D imaging techniques, such as Cone–Beam Computed Tomography (CBCT), provided a viable diagnostic aid to precisely assess the dimensions of the craniofacial complex in the three planes of the space, with a relatively low dose of radiation and limited costs. Due to shortened scan time and the high-resolution images, CBCT has been widely used for its beneficial contribution in orthodontic and TMJ diagnosis.

Although the clinical significance of the ideal condylar position in the glenoid fossa has been extensively questioned [8], gathering accurate information regarding TMJ anatomy provides deeper understanding in craniofacial morphology. Furthermore, it has been hypothesized that the shape and the size of the condyle play a primary role in the long-term stability of orthognathic therapies, due to the correlation between condylar morphology and masseter muscle development [9].

### Aim

The aim of this study was to three-dimensionally measure the condyle–fossa relationship, the condylar morphology and the facial asymmetry of patients with a different skeletal vertical growth pattern.

## 2. Materials and Methods

### 2.1. Sample

For the current retrospective study, ethical approval was obtained from the ethical committee of PDM university, Faculty of Dental Sciences, Bahadurgarh, India (ethical approval number PDMDCRI/2019/777, dt.19/12/2019). Diagnostic CBCT images of 56 patients (112 TMJs, age range 14–25 years) were collected from the archives of a radiographic diagnostic centre in Delhi (India). All CBCT scans were acquired as part of the planning stage for orthodontic treatment with an i-CAT Cone Beam 3D Dental Imaging system (i-CAT Classic, Imaging Sciences International, Hatfield, PA, USA). Each volumetric data set was acquired with a 20 s scan time with a 16 (diameter) and 22 (height) cm field of view and at a resolution of 0.25 mm voxels. All images were collected at 120 kVp and 5 mA based on the manufacturer’s specification.

Inclusion criteria were: CBCT taken with teeth in maximum intercuspation, no previous orthognathic surgery, no history of trauma, absence of complains associated with Temporomandibular Disorders (TMD). Subjects with congenital or systemic disease and severe mandibular deformity were excluded.

Using the Mandibular Plane (MP) angle (the angle formed by the intersection of the Frankfort horizontal plane and the mandibular plane), patients were classified into three groups: Hypodivergent (MP ≤ 23°; 20 subjects, 14 males, 6 females, mean age: 18.3 ± 5.6 years), Normodivergent (23° < MP < 30°; 20 subjects, 13 males, 7 females, mean age: 19 ± 5.6 years), and Hyperdivergent (MP ≥ 30°; 15 subjects, 8 males, 7 females, mean age 18.7 ± 5.2).

### 2.2. Measurements

**Figure 1:**
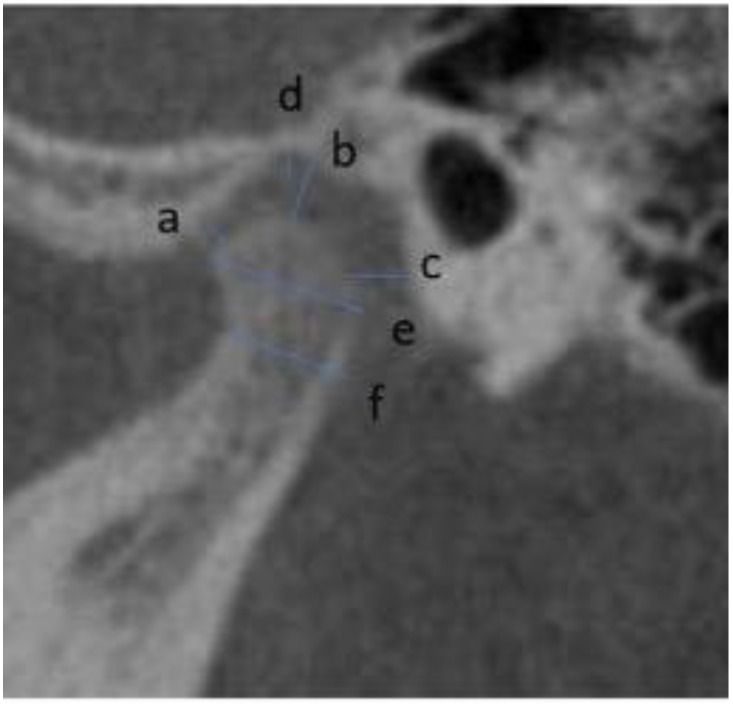
Sagittal view. a—anterior joint space; b—superior joint space; c—posterior joint space; d—depth of the glenoid fossa, e—condylar length; f—condylar neck width.

Landmarks and measurements adopted in the current study have been used in previous studies [10,11,12], and described in Supplementary Table S1 and Table S2. Briefly, the position of each condyle in the fossa, and the morphology of the fossa were determined on sagittal slices. In particular, the slices that showed the greatest anteroposterior dimension of the condylar head was selected [13]. The following measurements were recorded on the sagittal plane: anterior joint space (AS), superior joint space (SS) and posterior joint space (PS) and depth of the mandibular fossa. Furthermore, condylar length and condylar neck width were measured [14] (Figure 1).

**Figure 2:**
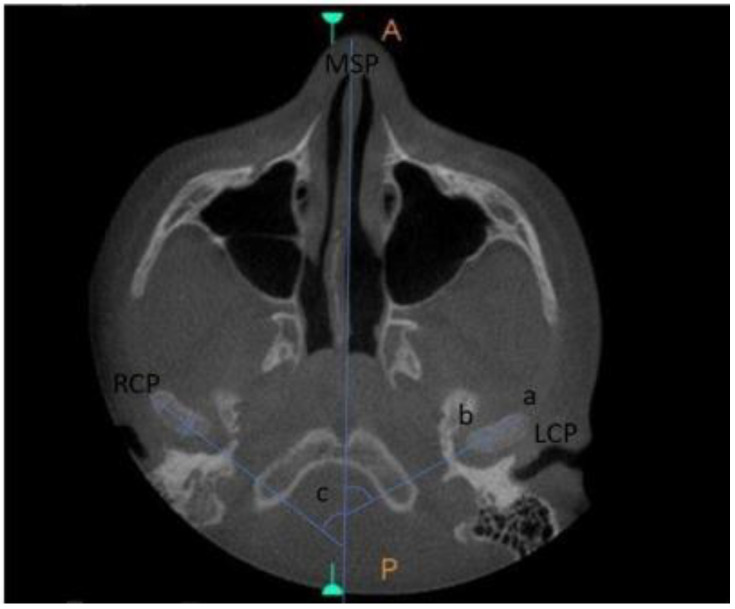
Axial view: a—mediolateral diameter of the condyle; b—antero posterior diameter of the condyle; c—condylar axis angle.

In the axial view, the slice showing the maximum mesiodistal diameter of the condyle was selected to measure the antero-posterior diameter of the condyle, the mediolateral diameter of the condyle, the condyle axis angle, the antero-posterior difference between the geometric centre of the right and left condylar processes as reflected on the Median Sagittal Reference Plane (MSP), and the distance between the geometric centres of the condylar processes and the MSP (Figure 2). The MSP was identified as the plane passing the line joining the Nasion point to the Basion point [15].

**Figure 3:**
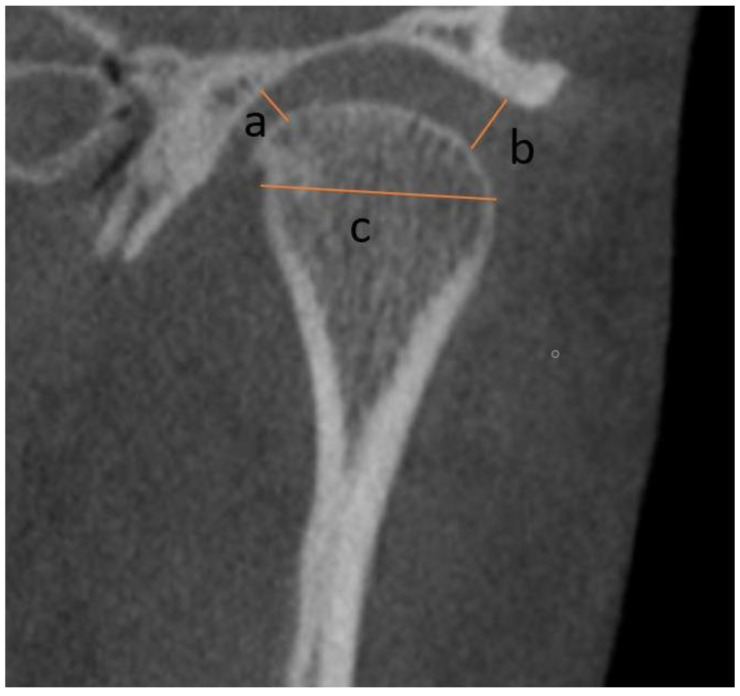
Coronal view. a—medial joint space; b—lateral joint space; c—mediolateral condyle thickness.

In the coronal view, the slice showing the maximum mesiodistal diameter of each condyle was selected to measure the lateral joint space (LS), the medial joint space (MS), and the mediolateral thickness of condyle (Figure 3).

**Figure 4:**
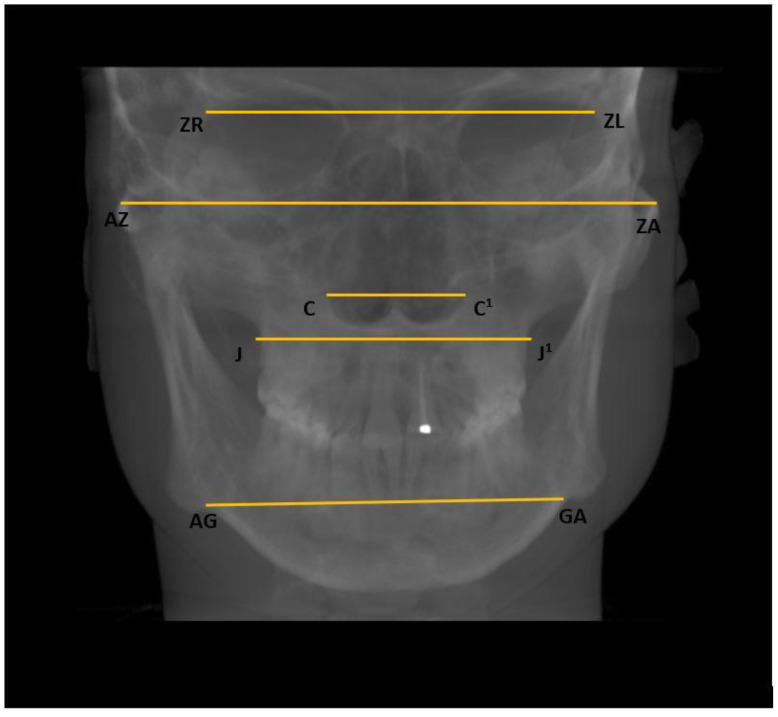
Horizontal measurements of facial asymmetry.

**Figure 5:**
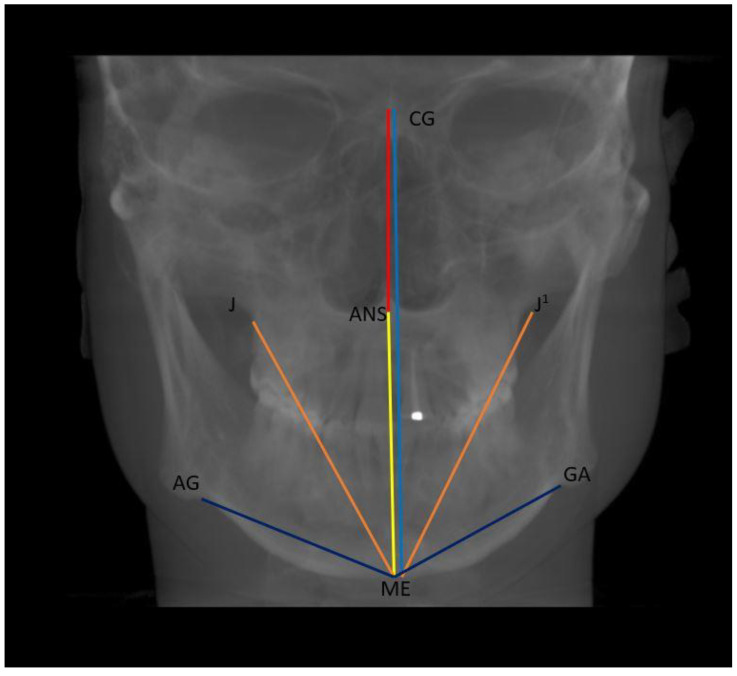
Vertical measurements of facial asymmetry.

**Figure 6:**
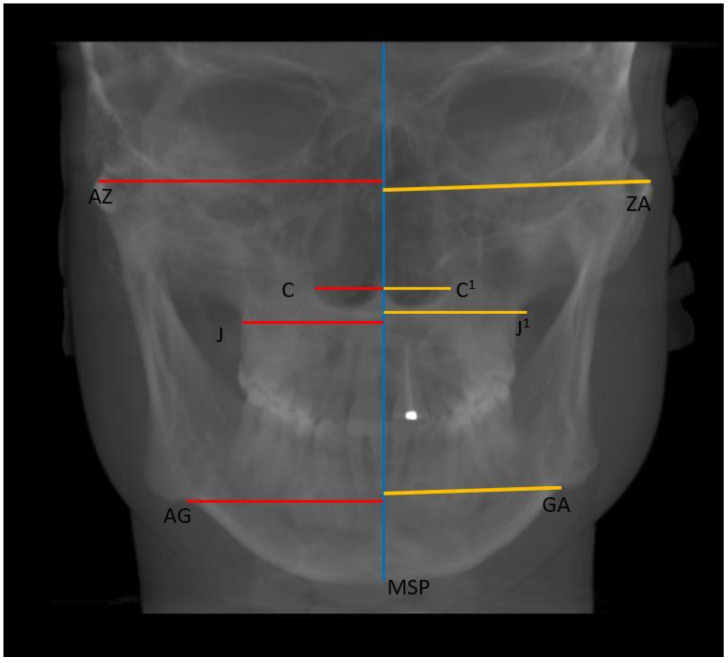
Asymmetry measurements on the MSP (Midsagittal Plane).

For the assessment of the facial asymmetry, both horizontal and vertical dimensions were measured:

- Horizontal: Nasal cavity width (C–C^1^), distance between zygomaticofrontal sutures (ZR–ZL), distance between the centres of the roof of the zygomatic arch (AZ–ZA), distance between the jugal processes (J–J^1^), and distance between the antegonial points (AG–GA) (Figure 4);

- Vertical: distance between Crista Galli to Menton (Cg–Me), distance between Anterior Nasal Spine and Menton (ANS–Me), distance between Crista Galli and Anterior Nasal Spine (Cg–ANS), distance between Jugal Process and Menton (J–Me left and right), distance between Antegonial notch and Menton (Ag–Me left and right) (Figure 5);

Finally, linear distances from left and right landmarks (AZ, C, J, and AG) to MSP, and differences in the vertical dimension of the perpendicular projections of bilateral landmarks to MSP were measured (Figure 6).

### 2.3. Statistical Analysis

The data were entered in Microsoft Excel 2007 and analysed using the IBM SPSS statistical software (Version 19.0). Continuous data were computed as means and standard deviations. One-way analysis of variance (ANOVA) was used to test the between-groups comparison, followed by post hoc analysis. A paired sample *t*-test was applied to check the within-group difference between right and left condyles. Statistical significance was set at *p* < 0.05.

## 3. Results

In the current retrospective sample, on the sagittal view, statistically significant difference between the three groups was observed for the right AS (*p* = 0.005), with greater values in the Hyperdivergent group, followed by the Normodivergent and the Hypodivergent groups (Table 1). Non significant differences were found among the three groups for all the other variables measured in the sagittal view (all *p* > 0.05). Significant differences between the right and left sides were observed only for the Condylar Length in the Normodivergent group and for the Condylar Neck Width in the Hypodivergent groups.

In the axial view, the mediolateral diameter of the condyle, of both the left and right side, was significantly smaller in the Hyperdivergent group, compared to the Normo- and the Hypodivergent groups (Table 2). No significant differences between the three study groups were observed for all the remaining variables on the axial plane (all *p* > 0.05). The distance between the geometric centre of the condyle and the MSP was significantly different between the left and right side in all the three groups, with right distances being greater than left distances.

In the coronal view, the left condyle of the Hyperdivergent group showed significantly smaller mediolateral thickness, as compared to the same side in the Normo- and Hypodivergent patients (Table 3). No significant differences among the three groups were observed for all the remaining variables assessed on the coronal plane (all *p* > 0.05). Furthermore, only in the Hyperdivergent group was the lateral joint space significantly different between the right and the left side.

Considering horizontal and vertical parameters for asymmetry, the antegonial distance was significantly larger in the Normodivergent group, as compared to the Hypo- and the Hyperdivergent patients (Table 4), while ANS-Me and Ag-Me (left and right) were greater in the Normodivergent group, followed by Hyper- and Hypodivergent patients (Table 5).

Among linear asymmetry values, only right side antegonial notch measurements showed statistically difference among groups (Table 6).

### Fi-Index Tool

This manuscript has been checked with the Fi-index tool [16,17] and obtained a score of 0.03 on 30 November 2022 according to Scopus^®^ for all authors. In this case, a low value has been obtained, the fact that it deviates from the value of 0 is justifiable by the fact that the articles cited in the text concern a bibliometric theme such as the manuscript.

## 4. Discussion

The present study aimed at three-dimensionally assessing positional and morphological features of the TMJs (condyle–fossa relationship and condylar morphology) and facial asymmetry of healthy adults divided according to different vertical skeletal growth patterns. The null hypothesis was that condylar position, condylar morphology and facial asymmetry did not differ significantly among hyperdivergent, normodivergent and hypodivergent patients. The statistical analysis revealed sporadic significances, often inconsistent between the left and right side in all three of the spatial dimensions.

Condyle position and morphology are extremely variable among individuals. Factors influencing the complex condyle–fossa anatomy include genetics, sex hormones, mechanical and functional load of the TMJ, and physiological constant remodeling. One previous study by Park and co-worker [18] addressed the three-dimensional position and morphology of the condyle according to different vertical skeletal patterns. The authors pointed out no significant differences in the anterior and posterior joint spaces among the groups, while a significantly smaller superior joint space was observed in the hyperdivergent group supporting the fact that the vertical skeletal pattern was associated with more superiorly positioned condyles. Furthermore, hyperdivergent facial morphology was associated with smaller antero-posterior and medio-lateral condyle widths as well as a narrower condyle head angle. One more recent study on the same topic pointed out a more anterior-position of the condyle in patients with high angle vertical patterns than in those with normal and low angle vertical patterns, while no significant differences were observed in the condylar position between low angle and normal angle subjects [19]. In the current study, a significant increase of the anterior joint space was observed in the hyperdivergent group, as compared to the normo- and to the hypodivergent groups, indicating a more posterior position of the condyle in hyperdivergent individuals. Furthermore, the medio-lateral diameter and medio-lateral thickness of the condyle resulted in being significantly reduced in the hyper-divergent group as compared to the normo- and hypodivergent groups, supporting smaller condyle dimensions in hyperdivergent individuals. One possible explanation for the discrepancy in the observed results is that, in both studies, the antero-posterior skeletal relationship was not considered. For instance, Song and colleagues [20] observed differences in condylar morphology, joint space, joint fossa morphology, and condylar position among different Angle classifications. Ma and co-authors [5] find out that participants in the group with Class II hyperdivergent patterns had a smaller and narrower condyle as compared with the Class III hyperdivergent, Class I hyperdivergent and Class I normodivergent subjects. Furthermore, among skeletal Class II female patients, high angle individuals show shorter condyle diameters, smaller glenoid fossa, flatter articular eminence and smaller superior and anterior joint space as compared to low angle patients [6]. In addition, Fan and co-workers observed, on CBCT of normodivergent individuals, significant differences in the TMJ osseous morphology between Class I and Class II patients [21]. Therefore, it seems that not only the vertical skeletal growth pattern, but also the sagittal maxillo-mandibular relationship might play a role in the different position and morphology of the TMJ condyle.

Interestingly, magnetic resonance imagining studies confirmed that high angle Class II individuals present more anteriorly positioned condyles, reduced anterior joint space and increased posterior joint space, and also pointed out a more anterior and mesial disc position, as compared to Class II horizontal cases [22]. These anatomical findings suggest that Class II hyperdivergent individuals might be more susceptible to the development of TMD. Similar findings were already reported in a previous systematic review, by Manfredini and co-workers, supporting increased frequency of disc displacement and degenerative joint disorders in the Class II profile with a hyperdivergent pattern of growth [23]. Hence, especially in Class II vertical individuals, it seems crucial to perform complete TMJ evaluation before the commencement of any type of orthodontic treatment in order to intercept and to manage TMD problems prior to orthodontics.

Studies on craniofacial patterns of TMD patient groups pointed out that joint disorder TMD patients had significantly more retropositioned mandibles and steeper mandibular planes when compared to myogenic TMD patients, who had normal anteroposterior and vertical craniofacial patterns [24]. Studies on the mechanical load of the TMJ comparing dolichofacial with brachyfacial volunteers demonstrated that dolichofacial subjects produced significantly larger TMJ loads as compared to brachyfacial subjects. Hence, large condyles are less susceptible to mechanical stress than small condyles, thus protecting against the subsequent onset of disc derangements [25,26]. In addition, a recent three-dimensional study reported an association between the presence of TMJ pain and a smaller condylar volume [27]. Therefore, condylar morphology and dimension, that are extremely variable among individuals, are to be considered potential risk factors for the development of articular disorders, more than the condylar position in the fossa. On top of that, it should be further underlined that current evidence supports the fact that orthodontic treatment cannot prevent or increase the risk of developing TMD, and orthodontists and general dentists should be aware about the multifactorial etiology of TMD and should be instructed regarding the available tools to manage patients before, during, and after any dental or orthodontic intervention [28].

The axial view of CBCT is considered the most accurate view to assess the symmetry and dimension of the condyles as it shows both condyles in the same image and allows for determining a reference plane such as the MSP. In this study, the distance between the geometric center of the condyle and the MSP was significantly different between the left and right side in all the three groups, with right distances being higher than left distances. Previous studies have suggested that there is a tendency for individuals with a hyperdivergent growth pattern to have more severe craniomandibular asymmetries [29,30], while a recent three-dimensional study pointed out no differences in the asymmetry indices between different vertical facial morphologies [31]. Therefore, the increased distance of the right condyle compared to the left condyle observed in the three study groups of the current sample could be due to chance or to the orientation of the head during the CBCT recording.

Different bi-dimensional methods, such as lateral cephalograms, have been used for the assessment for condyle–fossa morphology and position. For the current study, CBCT was used, which is currently considered the best approach to determine not only linear but also volumetric measurements of bony structures of TMJ [32].

It has to be underlined that, for the propose of the current study, TMD clinical diagnosis or history of TMD were excluded. Therefore, the current findings cannot be extended to patients’ populations as subject selection might be a drawback. Furthermore, limitations of the study include the determination of the measurement position and the lack of reliability assessment for repeated measurements. However, measurements were performed by one single expert operator in order to limit the measurements errors.

## 5. Conclusions

Among different vertical growth patterns in TMD free individuals, condylar dimension was significantly smaller in the hyperdivergent group, compared to normo- and hypo-divergent individuals. A more posterior position of the condyles in the fossa was also observed in high angle patients. No major differences were observed in terms of facial asymmetries among the three groups. Future studies are needed to elucidate whether differences of TMJ morphology are present in patients with TMD pain and/or dysfunction, in order to determine clinical implication of anatomical findings.

## Figures and Tables

**Table 1:** Right (R) and Left (L) sagittal variables, reported as means (mm) and Standard Deviations (SD) in the three study groups (Normo: Normodivergent; Hypo: Hypodivergent; Hyper: Hyperdivergent). Statistically significant *p*-values are reported in bold. Different letters indicate statistically significant difference between groups in the Tukey Post Hoc Test.

Group		AS			PS			SS			Condylar Length			Condylar Neck Width			Depth of Glenoid Fossa		
		R	L	*p*-Value R vs. L	R	L	*p*-Value R vs. L	R	L	*p*-Value R vs. L	R	L	*p*-Value R vs. L	R	L	*p*-Value R vs. L	R	L	*p*-Value R vs. L
Normo	Mean	1.99 a	1.98	0.065	2.75	2.65	0.448	3.07	6.89	0.790	6.74	3.10	0.010	7.07	6.96	0.594	1.17	1.53	0.801
	SD	0.59	0.79		0.89	0.81		0.80	1.34		1.51	0.76		1.37	1.36		0.35	0.23	
Hypo	Mean	1.69 a	1.78	0.841	2.88	2.98	0.203	3.33	7.37	0.850	7.21	3.38	0.055	7.40	7.57	0.047	1.25	1.08	0.699
	SD	0.51	0.69		0.99	1.39		1.37	1.44		1.43	1.31		1.18	1.44		0.38	0.23	
Hyper	Mean	2.43 b	1.98	0.577	2.79	2.55	0.731	3.24	6.11	0.643	6.46	3.11	0.202	7.18	7.31	0.195	1.29	1.23	0.493
	SD	0.84	0.70		0.96	0.84		1.02	1.79		1.73	0.69		2.22	1.48		0.29	0.29	
*p*-value ANOVA		**0.005**	0.610		0.900	0.440		0.740	0.060		0.330	0.610		0.590	0.390		0.580	0.240	

AS: Anterior Joint Space, PS: Posterior Joint Space; SS: Superior Joint Space.

**Table 2:** Right (R) and Left (L) axial variables, reported as means (mm or °) and Standard Deviations (SD) in the three study groups (Normo: Normodivergent; Hypo: Hypodivergent; Hyper: Hyperdivergent). Statistically significant *p*-values are reported in bold. Different letters indicate statistically significant difference between groups in the Tukey Post Hoc Test.

Group		AP Condyle Diameter			ML Condyle Diameter			Condylar Axis Angle			Condyle-MSP			AP Diff Condyle-MSP
		R	L	*p*-Value R vs. L	R	L	*p*-Value R vs. L	R	L	*p*-Value R vs. L	R	L	*p*-Value R vs. L	
Normo	Mean	5.91	6.04	0.008	18.69 a	18.08 a	0.509	68.76	73.25	0.304	47.53	41.51	**0.001**	8.00
	SD	0.59	1.29		2.17	2.13		10.37	8.45		4.48	7.25		4.09
Hypo	Mean	6.46	6.44	0.579	18.79 a	18.26 a	0.602	64.59	70.24	0.124	47.13	40.60	**0.001**	4.95
	SD	1.56	1.28		2.44	2.79		8.80	9.31		6.09	5.32		4.18
Hyper	Mean	5.57	5.72	0.024	15.75 b	15.46 b	0.752	63.54	73.47	0.098	47.83	40.17	**0.012**	6.67
	SD	1.77	1.59		1.79	2.35		9.15	11.56		6.77	4.53		4.49
*p*-value ANOVA		0.150	0.300		**0.010**	**0.002**		0.220	0.510		0.940	0.790		0.080

AP: Anteroposterior, ML: Mediolateral; MSP: Middle Sagittal Plane.

**Table 3:** Right (R) and Left (L) coronal variables, reported as means (mm or °) and Standard Deviations (SD) in the three study groups (Normo: Normodivergent; Hypo: Hypodivergent; Hyper: Hyperdivergent). Statistically significant *p*-values are reported in bold. Different letters indicate statistically significant difference between groups in the Tukey Post Hoc Test.

Group		LS			MS			ML Condyle Thickness		
		R	L	*p*-Value R vs. L	R	L	*p*-Value R vs. L	R	L	*p*-Value R vs. L
Normo	Mean	2.57	2.52	0.579	2.53	2.79	0.367	15.53	16.32 a	**0.013**
	SD	0.69	0.76		0.89	0.84		3.38	2.53	
Hypo	Mean	2.89	2.63	0.093	2.48	2.65	0.635	14.78	16.55 a	0.683
	SD	1.03	1.11		1.02	1.09		2.85	2.98	
Hyper	Mean	2.71	2.60	0.013	2.49	2.66	0.324	12.96	13.92 b	0.355
	SD	1.15	0.73		0.83	1.09		3.34	3.01	
*p*-value ANOVA		0.550	0.920		0.990	0.880		0.070	**0.020**	

LS: Lateral Joint Space; MS: Medial Joint Space; ML: Mediolateral.

**Table 4:** Asymmetry horizontal variables, reported as means (mm) and Standard Deviations (SD) in the three study groups (Normo: Normodivergent; Hypo: Hypodivergent; Hyper: Hyperdivergent). Statistically significant *p*-values are reported in bold. Different letters indicate statistically significant difference between groups in the Tukey Post Hoc Test.

Group		ZR-ZL	AZ-ZA	J-J1	AG-GA	C-C1
Normo	Mean	99.22	124.90	68.11	85.68 a	26.76
	SD	7.27	7.14	3.97	5.84	3.16
Hypo	Mean	100.16	123.58	68.31	80.69 b	25.89
	SD	5.41	6.59	5.55	5.41	2.19
Hyper	Mean	96.55	120.23	66.95	80.59 b	26.68
	SD	5.69	7.21	6.66	5.14	2.80
*p*-value ANOVA		0.230	0.150	0.740	**0.007**	0.540

**Table 5:** Asymmetry vertical variables, reported as means (mm) and Standard Deviations (SD) in the three study groups (Normo: Normodivergent; Hypo: Hypodivergent; Hyper: Hyperdivergent). Statistically significant *p*-values are reported in bold. Different letters indicate statistically significant difference between groups in the Tukey Post Hoc Test.

Group		Cg-Me	ANS-Me	Cg-ANS	J-Me		Ag-Me	
					R	L	R	L
Normo	Mean	113.13	63.13 a	50.54	70.41	68.32	49.57 a	44.39 a
	SD	5.64	5.67	4.67	6.48	5.67	5.59	5.52
Hypo	Mean	109.10	58.03 b	51.19	67.46	64.52	44.03 b	40.31 b
	SD	9.31	6.01	4.85	6.64	7.29	4.35	4.39
Hyper	Mean	111.09	62.08 a	49.86	70.29	68.01	48.41 a	43.76 a
	SD	6.96	5.33	3.45	6.75	6.69	3.51	4.02
*p*-value ANOVA		0.240	**0.020**	0.680	0.290	0.140	**0.001**	**0.020**

LS: Lateral Joint Space; MS: Medial Joint Space; ML: Mediolateral.

**Table 6:** Linear asymmetry variables, reported as means (mm) and Standard Deviations (SD) in the three study groups (Normo: Normodivergent; Hypo: Hypodivergent; Hyper: Hyperdivergent). Statistically significant *p*-values are reported in bold. Different letters indicate statistically significant difference between groups in the Tukey Post Hoc Test.

Group		Az-MSP		C-MSP		J-MSP		Ag-MSP	
		R	L	R	L	R	L	R	L
Normo	Mean	63.96	58.89	13.82	12.23	35.80	31.73	45.47 a	39.48
	SD	5.74	4.42	2.51	1.33	3.58	3.24	4.44	3.37
Hypo	Mean	64.24	59.64	13.25	12.49	35.43	32.19	42.58 b	37.99
	SD	3.95	3.71	2.10	1.45	3.49	2.69	3.72	3.62
Hyper	Mean	62.21	57.68	13.20	13.09	34.66	31.30	42.88 b	37.72
	SD	4.11	5.59	1.55	1.82	4.04	4.20	3.53	3.88
*p*-value ANOVA		0.410	0.450	0.610	0.250	0.660	0.630	**0.040**	0.280

## Data Availability

Not applicable.

## Supplementary Materials

The following supporting information can be downloaded at: https://www.mdpi.com/article/10.3390/ijerph20021437/s1, Table S1: Description of the linear and angular measurements of condyle morphology and position; Table S2: Description of landmarks for the assessment of asymmetry.
