# Supplementary material for: Three-Dimensional Assessment of Temporomandibular Joint Morphology and Facial Asymmetry in Individuals with Different Vertical Skeletal Growth Patterns
Source: Int J Environ Res Public Health. 2023 Jan 12;20(2):1437. doi: 10.3390/ijerph20021437 (PMC9859447; doi:10.3390/ijerph20021437)
Supplement: Supplementary file 1 [file ijerph-20-01437-s001.zip › ijerph-2023915-supplementary.pdf]

Table S1. Description of the linear and angular measurements of condyle morphology and position.

Measurement	Description
Anterior joint space (mm) [AS]	The shortest distance between the posterior wall of the articular tubercle and the most anterior point of the condylar head
Superior joint space (mm) [SS]	The distance between the most superior point of the mandibular fossa and the most superior point of the condylar head
Posterior joint space (mm) [PS]	The shortest distance between the posterior wall of the mandibular fossa and the most posterior point of the condylar head
Condylar length (mm)	Distance between the most anterior point of the condyle and the most posterior point of the condyle on sagittal plane
Condylar neck width (mm)	Distance between the most anterior point and the most posterior point of the condylar neck on the sagittal plane
Depth of the glenoid fossa (mm)	The distance between the most superior point of the mandibular fossa and the plane formed by the most inferior points of the articular tubercle and the post glenoid process
Antero-posterior [AP] condyle diameter (mm)	Distance between the most anterior point of the condyle and the most posterior point of the condyle on axial plane
Medio-lateral [ML] condyle diameter (mm)	Distance between the most lateral point of the condyle and the most medial point of the condyle on axial plane
Condylar axis angle (°)	Angle between the medio-lateral plane of the condylar process and the MSP
Medial Joint space (mm) [MS]	The distance between the most medial point of the mandibular fossa and the most medial point of the condylar head
Lateral Joint space (mm) [LS]	The distance between the most lateral point of the mandibular fossa and the most lateral point of the condylar head
Medio-lateral [ML] condyle thickness	Distance between the most lateral point of the condyle and the most medial point of the condyle on coronal plane

Table S2. Description of landmarks for the assessment of asymmetry.

Landmark	Abbreviation	Description
zygomaticofrontal sutures right (ZR) and left (ZL)	ZR / ZL	The centre of the cranial suture between the zygomatic bone and the frontal bone
zygomatic arch right (AZ) and left (ZA)	AZ / ZA	The centre of the roof of the zygomatic arch
jugal processes right (J) and left (J1)	J / J1	The point at the intersection of outline of maxillary tuberosity and zygomatic buttress
antegonial right (AG) and left (GA)	AG / GA	Left and Right point of deepest concavity between anterior convexity point and inferior gonion
nasal concha right (C) and left (C1)	C/C1	Left and Right attachment of the inferior turbinate
crista galli	Cg	Upper part of the perpendicular plate of the ethmoid bone of the skull.
menton	Me	The lowest point of the mandibular symphysis
anterior nasal spine	ANS	Tip of the median sharp bony process of the palatine bone in the hard palate
